# “You cannot stay with one person once you begin having sex at a young age”: the prevalence, correlates and effects of early sexual debut among children in Ghana

**DOI:** 10.1186/s12978-024-01775-4

**Published:** 2024-03-23

**Authors:** Frank Kyei-Arthur, Martin Wiredu Agyekum, Sylvester Kyei-Gyamfi

**Affiliations:** 1 Department of Environment and Public Health, University of Environment and Sustainable Development, Somanya, Ghana; 2 Institute for Educational Research and Innovation Studies, University of Education, Winneba, Ghana; 3 Department of Children, Ministry of Gender, Children and Social Protection, Accra, Ghana

**Keywords:** Early sexual debut, Children, Prevalence, Causes, Effects, Correlates, Ghana

## Abstract

**Background:**

Children’s initiation of early sex has several negative implications on their sexual and reproductive health, growth and development. In Ghana, few studies on early sexual debut have focused on adolescents. Therefore, this study examined the prevalence, causes, correlates and effects of early sexual debut among children aged 8 to 17 in Ghana using secondary data from the Department of Children of the Ministry of Gender, Children, and Social Protection.

**Methods:**

A convergent parallel mixed-method approach guided the study. Descriptive statistics and multivariable binary logistic regression were used to analyse the quantitative data, while thematic analysis was used to analyse the qualitative data.

**Results:**

The study found that the prevalence of early sexual debut among children was 13.2%, which is more predominant among female children. The main causes of early sexual debut include engaging in sex after watching pornography, self-desire to have sex, and being influenced by alcohol consumption. Also, age, sex, education, marital status, religion, ecological zone, living arrangements, and access to the Internet were significant correlates of early sexual debut. Early sexual debut increases children’s risk of unwanted pregnancy, which leads to the termination of children’s education or induced abortion. Also, early sexual debut had adverse impacts on the wellbeing of pregnant children and increased children’s risk of multiple lifetime sexual partners.

**Conclusions:**

This study demonstrated that socio-demographic characteristics of children (e.g., age, sex, education, and marital status) were significant correlates of early sexual debut. Policymakers need to design appropriate interventions, considering the socio-demographic characteristics of children, to curb its occurrence in Ghana.

## Background

Children’s engagement in sexual activity is a significant public health issue [1, 2], which is detrimental to children’s sexual and reproductive health, growth and development. Early sexual debut is defined as having had first sexual intercourse at or before age 14 years of age [3, 4]. Although it is the age at which people engage in their first sexual activity, the age at first sexual experience varies from place to place, between individuals, and is influenced by various circumstances [3]. Even though research has shown that having sex is incredibly healthy and can activate several neurotransmitters that affect human brains and other human organs [5], having sex at a very young age may be harmful [1, 6].

Early sexual debut has several negative implications on young people’s sexual and reproductive health. The effects include a rise in the number of lifetime sexual partners, unprotected sexual intercourse, sexually transmitted infections, including HIV/AIDS, unwanted and unintended pregnancies, and unsafe abortions [1, 3, 7–9]. Due to the adverse health outcomes of early sexual debut, many countries have made laws to set the age of sexual consent. In Ghana, the legal minimum age of sexual consent is 16 [10]. Section 101 of the Criminal Code 1960 (Act 29), which was amended in 1998 by the Criminal Code (Amendment), Act 554, stipulates the legal age of sexual consent in Ghana at 16 years [10].

Even though there are laws regulating sexual consent, many children in Ghana start having sexual relationships at very young ages. According to the 2018 Multiple Indicator Sampling Survey (MICS), 7% of adolescent boys and 11% of adolescent females aged 15 to 24 started having sex before turning 15 [11]. Thus, these adolescents are engaged in unlawful sexual activities because of the age at which they engaged in those sexual activities. In Ghana, sex with or without the consent of a person under 16 years constitutes defilement. Such sexual act is unlawful, and anyone found guilty faces a sentence of 7 to 25 years in prison [12]. Notwithstanding Ghana’s 16-year-old sexual consent law, there is increased evidence of defilement cases across the country, although these cases are often underreported [13–15].

In Ghana and other sub-Saharan African countries, issues related to sexual intercourse are rarely discussed with children due to varied factors, including culture and religious beliefs [16–19]. Although there are studies on early sexual debut in Ghana, these studies have some limitations. First, these studies have focused on the youth [20, 21] and women, especially those in their reproductive ages (15–49) [22, 23]. Second, these studies have emphasised the correlates of early sexual debut [20–23]. For instance, Alhassan et al.’s [23] study among Ghanaian women aged 15–49 found that age, education, place of residence, household wealth, marital status, substance and alcohol use, contraceptive use, heard of HIV and Internet use, among others were significant correlates of early sexual debut. Similarly, Asante et al.’s [20] study found that age, education, place of residence, contraceptive use, access to media, and household wealth were significant correlates of early sexual debut. Third, few studies have focused on adolescents [1, 24, 25]. However, most of these studies focused on female adolescents and used only quantitative methods [24, 25]. Hence, this study focused on both male and female children using a mixed-method approach to contribute to the early sexual debut literature in Ghana and the world at large.

Understanding the causes and effects of early sexual debut and the correlates of early sexual debut are essential for guiding policy development, effective programming, and empowering adolescents to reduce the incidence of early sexual debut and its adverse consequences. Therefore, this study aimed to examine the prevalence, causes, correlates and effects of early sexual debut among children in Ghana.

## Methods

### Study design and sampling procedure

This study used secondary data from the Department of Children of the Ministry of Gender, Children, and Social Protection (MoGCSP). The data covered varied topics on children’s protection and general wellbeing, such as children’s rights, internet use, living arrangements, health, and employment. It was designed based on the convergent parallel mixed-method approach. A convergent parallel mixed-method approach facilitates the concurrent collection and analysis of quantitative and qualitative data [26]. A multi-stage sampling technique was used to select respondents (children aged 8 to 17) for the quantitative data. First, 20% of 216 districts in Ghana as of 2018 were selected based on child protection and wellbeing issues, such as child marriage and labour. This resulted in the selection of 43 districts. Second, 15 enumeration areas were selected in each of the 43 districts, which resulted in the selection of 645 enumeration areas. Third, children aged 8 to 17 in households in each enumeration area were selected to participate in the study. Only one child aged 8 to 17 could be interviewed in each household. In total, 5024 children aged 8 to 17 were interviewed across the country.

Regarding the qualitative data, ten focus group discussions (FGDs) were conducted with children aged 8 to 17 at convenient places. The FGDs were made up of between 7 and 14 participants, which was segmented by the sex of participants. Also, fifty expert interviews were conducted with officials of state agencies mandated to promote and protect children’s rights and wellbeing, such as officials of Commission on Human Rights and Administrative Justice; Department of Gender, MoGCSP; Ghana Health Service; Ghana Education Service; and Metropolitan, Municipal and District Assemblies of Amansie West, Dormaa, Komenda-Edina-Eguafo-Abirem, Asuogyaman, Tema, Yendi, Kassena-Nankana, Jirapa, Biakoye, and Prestea-Huni Valley. More details about the sampling procedure can be found in previous studies [27, 28].

### Study setting

Ghana is a lower-middle income country in West Africa, bounded to the North by Burkina Faso, the South by the Gulf of Guinea, the East by Togo, and the West by Côte d’Ivoire. In 2018, Ghana had ten regions. However, there are currently 16 regions in Ghana. As of 2021, Ghana had a population of 30,832,019 [29].

### Description and conceptualisation of variables

#### Dependent variable

The dependent variable for the study was early sexual debut. Two questions were used to measure early sexual debut. During the quantitative data collection, children were asked, “Have you ever had any sexual experience?” and “At what age did you have your first sexual experience?”. The second question was answered by children who indicated they had sexual experience. Children who had ever had sex before or at 14 years were classified as initiating sex early. For this study, sexual experience was defined as any sexual activity that causes sexual arousal, and it includes anal and vaginal intercourse, oral-genital contact, kissing, touching, and fondling.

#### Independent variables

The independent variables were sex (male and female), age (8–10 years, 11–13 years, and 14–17 years), education (no education, primary, Junior High School (JHS), and Senior High School (SHS)/Vocational/Technical/Commercial, and Tertiary), marital status (married and not married), religion (Christianity, Islam, and Other). Other independent variables include living arrangements (both biological parents, biological mother alone, biological father alone, and others) and access to the Internet (no and yes). The region of residence was recategorised as ecological zones, which had three categories: Coastal (Western, Central, Greater Accra, and Volta regions), Middle (Eastern, Ashanti and Brong Ahafo regions), and Northern (Northern, Upper East and Upper West regions) zones.

### Data collection

The data collection took place in April and October 2018. Children aged 8 to 17 and state agency officials mandated to promote and protect children’s rights and wellbeing were interviewed. Experienced research assistants were recruited and had a day of training on the quantitative and qualitative data collection instruments.

The National Child Protection Committee (Institutional Review Board) of the Department of Children of the Ministry of Gender, Children, and Social Protection (MoGCSP) approved the collection of the secondary data used for this study. Research assistants sought permission from the children and their parents/guardians included in the study. The purpose of the study, the general objectives, benefits, and risks of taking part in the study were explained by research assistants to the children and the parents/guardians. After that, written consent was sought from the parents/guardians and their children to participate in the study. Furthermore, participants were informed about strict confidentiality and anonymity of the information they would provide. Research assistants informed parents/guardians and their children about their right to stop participating in the study at any point if they desired to do so.

### Data analysis

Frequencies and percentages were used to describe the socio-demographic characteristics of children. Pearson’s chi-square test and Fisher’s exact test were used to examine the association between children’s early sexual debut and their socio-demographic characteristics and the causes of early sexual debut among children and the sex of children. A multivariable binary logistic regression was performed to examine the correlates of early sexual debut. The Statistical Package for the Social Sciences (SPSS) version 26 was used to perform all statistical analyses. At the 95% confidence interval, variables were determined to be statistically significant.

All interviews were recorded and afterwards transcribed word-for-word from the local languages the interviews were conducted (including Fante, Bono, and Gurune) into English. The qualitative data were analysed thematically using NVivo version 10 software. All transcripts were read superficially to get an overview of respondents’ perspectives on children’s sexual experiences. Afterwards, each transcript was read thoroughly, and statements relevant to the children’s sexual experiences were assigned codes. Sub-themes were developed from clusters of similar codes discovered in transcripts. Furthermore, similar sub-themes were clustered to form themes.

## Results

### Socio-demographic characteristics of respondents

Table 1 describes respondents’ socio-demographic characteristics. A little over half of the children (51.1%) were males, while about 3 out of 10 were aged 11 to 13 (29.8%). A higher proportion of children were Christians (75.1%), had primary education (42.1%) and resided in the Middle ecological zone (40.7%). Also, more than half of the children (57.4%) lived with both biological parents. The majority of children (93.3%) had access to the Internet.

**Table 1:** Socio-demographic characteristics of respondents by early sexual debut

Variables	Freq. (%)	Early sexual debut		
		No	Yes	
		Freq. (%)	Freq. (%)	*P*-value
*Sex*				
Male	2566 (51.1)	2295 (89.4)	271 (10.6)	0.000
Female	2458 (48.9)	2066 (84.1)	392 (15.9)	
*Age*				
8–10	1330 (26.5)	1311 (98.6)	19 (1.4)	0.000
11–13	1497 (29.8)	1453 (97.1)	44 (2.9)	
14–17	2197 (43.7)	1597 (72.7)	600 (27.3)	
*Education*				
No education	27 (0.5)	27 (100.0)	0 (0.0)	0.000*
Primary	2116 (42.1)	2083 (98.4)	33 (1.6)	
JHS	1584 (31.5)	1441 (91.0)	143 (9.0)	
SHS/Voc./Tech./Com.	1183 (23.5)	756 (63.9)	427 (36.1)	
Tertiary	114 (2.3)	54 (47.4)	60 (52.6)	
*Marital status*				
Married	47 (0.9)	17 (36.2)	30 (63.8)	0.000
Not married	4977 (99.1)	4344 (87.3)	633 (12.7)	
*Religion*				
Christianity	3771 (75.1)	3303 (87.6)	468 (12.4)	0.000
Islam	1083 (21.6)	927 (85.6)	156 (14.4)	
Other	170 (3.4)	131 (77,1)	39 (22.9)	
*Ecological zone*				
Coastal	1806 (35.9)	1497 (82.9)	309 (17.1)	0.000
Middle	2043 (40.7)	1806 (88.4)	237 (11.6)	
Northern	1175 (23.4)	1058 (90.0)	117 (10.0)	
*Living arrangements*				
Both biological parents	2883 (57.4)	2600 (90.2)	283 (9.8)	0.000
Biological mother alone	987 (19.6)	837 (84.8)	150 (15.2)	
Biological father alone	225 (4.5)	181 (80.4)	44 (19.6)	
Other	929 (18.5)	743 (80.0)	186 (20.0)	
*Access to the Internet*				
No	4688 (93.3)	4121 (87.9)	567 (12.1)	0.000
Yes	336 (6.7)	240 (71.4)	96 (28.6)	
**Total**	**5024 (100.0)**	**4361 (86.8)**	**663 (13.2)**	

* Fisher Exact Test

### Prevalence of early sexual debut

From Table 1, 13.2% of children initiated sex early (before or at age 14). More female children (15.9%) initiated sex early than male children (10.6%). Older children (14–17 years) (27.3%) significantly initiated sex early than children aged 8 to 10 (1.4%) and 11 to 13 (2.9%). A higher proportion of children with tertiary education (52.6%) initiated sex early than children with other educational attainment. In addition, a higher proportion of married children (63.8%) commenced sex early than unmarried children (12.7%).

Regarding religion, a higher proportion of children with other religion category (22.9%) significantly initiated sex early than children who were Muslim (14.4%) and Christians (12.4%). More children who resided in the Coastal zone (17.1%) significantly initiated sex early than children from the Middle (11.6%) and Northern (10.0%) zones. Also, children who lived with neither of their biological parents (20.0%) significantly initiated sex early than children who lived with either father alone (19.6%) or mother alone (15.2%) and both parents (9.8%). A higher proportion of children who had access to the Internet (28.6%) significantly initiated sex early than those without Internet access (12.1%).

### Causes of early sexual debut

Table 2 shows the causes of early sexual debut among children by sex. Children were asked to explain the factors that led to their early sexual debut. For male children, the main causes of early sexual debut were being influenced by alcohol consumption (27.5%), self-desire to experience sex (24.9%) and feeling like having sex after watching pornography (24.5%) to engage in early sexual debut. About 12% of male children exchanged hetererosexual sex for academic assistance.

**Table 2:** Causes of early sexual debut among children by sex

Sex of children	Causes of early sexual debut					
	Watched porn and felt like trying/curiosity	Self-desire to experience sex	Under the influence of alcohol during a social event	For financial assistance	Exchange sex for academic assistance	Marital relationship
	Freq. (%)	Freq. (%)	Freq. (%)	Freq. (%)	Freq. (%)	Freq. (%)
Male	66 (24.5)	67 (24.9)	74 (27.5)	22 (8.2)	31 (11.5)	9 (3.3)
Female	101 (26.0)	87 (22.4)	64 (16.5)	57 (14.7)	43 (11.1)	37 (9.5)
Total	167 (25.4)	154 (23.4)	138 (21.0)	79 (12.0)	74 (11.2)	46 (7.0)
*P*-value	0.000					

For female children, the main causes of early sexual debut were feeling like having sex after watching pornography (26.0%), self-desire to experience sex (22.4%), and being influenced by alcohol consumption (16.5%). About 15% of female children mentioned financial assistance made them initiate sex early. The association between sex and causes of early sexual debut was statistically significant (*p*-value = 0.000).

From the qualitative data, FGDs with children confirmed sex differentials in the causes of early sexual debut among children. FGDs with children in Tema Community 1 in the Coastal ecological zone highlighted that some children, especially female children, are compelled to have sex in return for their friends helping them with their schoolwork. A participant had this to say:

*Several of my classmates have been bragging that they have sex with certain girls because they want their help to do their schoolwork. This typically happens when Seniors and Juniors interact about schoolwork and the boys discover that some girls need assistance to accomplish their work. Relationships between Senior boys and Junior girls are common in the local schools. (FGD 1)*

In Saboba in the Northern ecological zone, children explained that many female children had their first sexual experience in their betrothal marriage, where they are expected to engage in sexual intercourse with their partners. A key informant reported the following:

*Many of the girls here had sex very early in life because they were married while they were children, and once married, they were required to consummate the union by having sex with their husbands. Sadly, many girls are far below the legal age of sexual consent in the country. (KII 1)*

Key informants narrated that some children are exposed to pornographic or sexually explicit materials on their phones, influencing them to initiate sex early since they want to experiment with what they watch. This phenomenon is common among male and female children residing in both urban and rural areas. Below is the narrative of a key informant:

*In comparison with children who have never viewed or read sexually explicit materials, children who watch sexual content are more likely to have early sex. As it is simple to get on the Internet, many watch pornography on their phones. Once they get it [pornography], they share it and watch it among themselves. When they view them, they desire to try out what they watch. Many become curious after watching pornography, and some engage in early sex.* (KII 2)

In addition, key informants indicated as adolescents age, they become knowledgeable about sex, and they, therefore, naturally want to engage in sexual intercourse to experience how sex feels like.

*When the children reach 14, 15 and 16, they become curious about sex since they may have heard about it from their friends, seen sexual scenes in movies, read about them, and so they are curious to experience it. There is a natural inclination to try it to see how sex feels, so it becomes difficult to stop them when the opportunity arises* (KII 3).

### Correlates of early sexual debut

Regarding education, no education category had few responses, so it was merged with primary education for the multivariable analysis. From the multivariable binary logistic regression analysis (Table 3), female children (AOR = 1.594, 95% C.I. = 1.312–1.938) were more likely to initiate sex early than male children. Children with tertiary (AOR = 21.241, 95% C.I.= 9.747–46.285), SHS/Voc./Tech./Com. (AOR = 9.080, 95% C.I.= 4.572–18.032), and JHS (AOR = 2.509, 95% C.I.= 1.316–4.784) had higher odds of initiating sex early than those with no education/primary education. Regarding religion, children who belonged to the other religion category (AOR = 2.397, 95% C.I.= 1.531–3.753) and those who were Moslems (AOR = 1.988, 95% C.I.= 1.531–2.581) were more likely to initiate sex early than children who were Christians. Children who lived with their biological father alone (AOR = 1.518, 95% C.I.= 1.009–2.283), the biological mother alone (AOR = 1.400, 95% C.I.= 1.096–1.788), and non-parents (AOR = 1.316, 95% C.I.= 1.040–1.666) had higher odds to initiate sex early than children who lived with both biological parents. Children who had access to the Internet were 1.668 times (AOR = 1.668, 95% C.I.= 1.245–2.234) more likely to initiate sex early than children who had no access to the Internet.

**Table 3:** Correlates of early sexual debut

	AOR	95% C.I.	*P*-value
*Sex*			
Male (RC)			
Female	1.594	1.312–1.938	< 0.001
*Age*			
8–10	0.279	0.123–0.629	0.002
11–13	0.314	0.207–0.478	< 0.001
14–17 (RC)			
*Education*			
No education/Primary (RC)			
JHS	2.509	1.316–4.784	0.005
SHS/Voc./Tech./Com.	9.080	4.572–18.032	< 0.001
Tertiary	21.241	9.747–46.285	< 0.001
*Marital Status*			
Married (RC)			
Not married	0.164	0.078–0.341	< 0.001
*Ecological zone*			
Coastal (RC)			
Middle	0.668	0.542–0.825	< 0.001
Northern	0.456	0.341–0.610	< 0.001
*Religion*			
Christianity (RC)			
Islam	1.988	1.531–2.581	< 0.001
Other	2.397	1.531–3.753	< 0.001
*Living arrangements*			
Both biological parents (RC)			
Biological mother alone	1.400	1.096–1.788	0.007
Biological father alone	1.518	1.009–2.283	0.045
Other	1.316	1.040–1.666	0.022
*Access to the Internet*			
No (RC)			
Yes	1.668	1.245–2.234	0.001

*AOR Adjusted odds ratio, C.I. Confidence interval*

Children aged 8 to 10 (AOR = 0.279, 95% C.I.= 0.123–0.629) and aged 11 to 13 (AOR = 0.314, 95% C.I.= 0.207–0.478) were less likely to initiate sex early than children aged 14–17. Similarly, unmarried children (AOR = 0.164, 95% C.I.= 0.078–0.341) were less likely to initiate sex early than married children. Children who resided in the Northern (AOR = 0.456, 95% C.I.= 0.341–0.610) and Middle (AOR = 0.668, 95% C.I.= 0.542–0.825) zones had lower odds of initiating sex early than those who resided in the Coastal zone (Table 3).

### Effects of early sexual debut

During the focus group discussions, three themes emerged as the consequences of early sexual debut: (a) risk of unwanted/unexpected pregnancy and associated consequences, (b) adverse impact on pregnant children’s wellbeing, and (c) risk of multiple lifetime sexual partners and its associated risks.

### Risk of unwanted/unexpected pregnancy and associated consequences

In all the FGDs, participants indicated that early sexual intercourse is often unprotected, leading to unwanted/unexpected pregnancy, which affects the education of females more than males. Participants explained that most female children in their communities who initiated sex early often get pregnant, eventually leading to the truncation of their education.

*Teenage pregnancy is prevalent in my community since many girls engage in sexual intercourse at younger ages, and they do not practice safe sex, which results in pregnancy. Many of them terminate their education because they cannot stand their colleagues’ mockery about their pregnancy in school. (FGD 2).*

Participants also highlighted that unwanted pregnancies among female children often result in induced abortions. A participant shared her experience below:

*I missed my period [menses] in SHS because I had sex with my boyfriend. After two weeks, I told him about it, and he suggested I use akpeteshie [a local alcoholic beverage] to terminate it. He offered me some pills to terminate the same pregnancy after the akpeteshie failed to work. But I refused to take the pills. Even though I did not terminate my pregnancy, many girls in my neighbourhood and others use various methods to terminate their unwanted pregnancies. (FGD 3).*

### Adverse impact on pregnant children’s wellbeing

Some participants remarked that unwanted pregnancies due to early sex initiation are undesirable for children’s general wellbeing, especially when the pregnancy is between two minors. Some parents eject their children from home as they feel disappointed by unwanted pregnancies. These ejected pregnant children independently have to take care of themselves and their unborn children, which can adversely impact the general wellbeing of the pregnant children and their unborn children. A participant in the FGD narrated that:

*Girls who experience unwanted pregnancy due to early sex initiation may face ejection from home by their parents because their parents sometimes feel betrayed and let down. Finding work to support themselves and their unborn child is necessary for such children. This often involves enduring hardships during pregnancy and childbirth as pregnant children must secure employment to care for themselves until delivery. (FGD 4).*

### Risk of multiple lifetime sexual partners and its associated risks

Early sex initiation may increase people’s number of sexual partners during their lifetime and may increase their risk of sexually transmitted infections (STIs). Participants reported that many of the children who initiate sex early tend to have several sexual partners over their lifetime, which increases their risk of STIs. One participant argued that:

*You cannot stay with one person once you begin having sex at a young age. If you are a girl, you will not be pleased with any man who does not fulfil your [sexual] needs, and you will be forced to obtain these [sexual] needs from other men. The same is true for boys who engage in sexual activity at a young age. They move from one girl to another, sleeping [having sex] with whoever they can get, eventually getting STIs like gonorrhoea and HIV. (FGD 5).*

Some participants revealed that most children who initiate sex early tend to become disrespectful. They cease to respect adults because, after having sex, they perceive they are mature enough to share the same space with adults. One participant indicated:

*The moment little children engage in sex, their attitudes and behaviour towards adults change for the worse. At home, they disrespect their parents, and in school, they exhibit similar traits of insolence to school authorities. (FGD 6).*

## Discussion

In this study, we examined the prevalence, causes, correlates and effects of early sexual debut among children in Ghana. The study found that 13.2% of children had sex early. The prevalence of early sexual debut reported in this study is lower than those found in other Ghanaian studies [20, 22, 23]. In these studies, the prevalence of early sexual debut were 26.7% [22], 32.5% [20], and 56.9% [23]. The differentials in the prevalence of early sexual debut could be due to differences in the study population and the definition of sexual debut. For instance, Asante et al.’s [20] study was conducted among youth aged 15 to 24 and early sexual debut was defined as sexual intercourse before or at 16 years. Also, Alhassan et al.’ [23] study was conducted among women in their reproductive years (15–49 years).

Although the prevalence of early sexual debut was low (13.2%) in this study, it is worrying since any sexual activity, with or without consent, with a child under 16 is considered defilement in Ghana [30]. This situation calls for state agencies mandated to promote and protect children’s rights and wellbeing to strengthen their advocacy to help reduce the prevalence of early sexual debut in Ghana.

Our findings highlighted the feeling of having sex after watching pornography, self-desire to experience sex, and being influenced by alcohol consumption were the main causes of early sexual debut among children in Ghana. Studies have found a link between watching pornography and having sexual experiences at a young age [31–33]. Also, studies have associated alcohol consumption with risky sexual behaviour [34, 35]. It is, therefore, unsurprising that alcohol consumption and watching pornography influence children to initiate early sex. Also, the quantitative and qualitative findings support the notion that some children had to have their first sex by exchanging sex for financial or academic assistance in school [36, 37].

Also, the study found that sex, age, education, marital status, religion, ecological zone, living arrangement, and access to the Internet were significant correlates of early sexual debut. Female children had higher odds of initiating sex early than male children. This finding supports earlier studies, which found females are more likely to initiate sex early than males [4, 21, 38]. Factors driving this phenomenon include early marriage, transactional sex, poverty and coercive sex [39, 40]. According to Awusabo-Asare et al. [40], female children are more likely to be forced into having sex than male children. Mufune [41] revealed that young females who try to refuse sex from their male partners might receive beatings.

With age, older children had higher odds of initiating sex early than younger children. This finding aligns with previous studies [42, 43], which demonstrated that as children get older, they initiate more sexual contact. Early sexual debut increases with an increase in educational level. Children with tertiary education had higher odds of initiating sex early than children with no education/primary. This finding is expected since the likelihood of children being older and having initiated sexual contact increases with increased educational level. However, this finding does not support previous studies, which found increased educational level as a protective factor against early sexual debut [20, 23, 44].

In terms of marital status, unmarried children had lower odds of initiating sex early than married children. Married children must engage in sexual intercourse with their partners, so they are expected to have higher odds of initiating sex early. In addition, children who belonged to the other religion category and those who were Moslems were more vulnerable to early sexual debut than children who were Christians. The practice of child marriage may explain why Muslim children have higher odds of initiating sex early than Christian children. Studies have documented a link between Islam and the practice of child marriage [45, 46]. However, studies have found Christianity as a protective factor against child marriage [46, 47].

Children who resided in the Northern and Middle zones had lower odds of initiating sex early than those who lived in the Coastal zone. In Ghana, teenage pregnancy is prevalent in the Coastal ecological zones [48, 49], so, unsurprisingly, children residing in the Coastal ecological zone have higher odds of initiation of early sex.

Regarding living arrangements, living with both biological parents was a protective factor against early sexual debut. This finding supports Roman Lay et al.’s [50] study in Brazil, which found that children living with both parents had lower odds of initiating early sex. Living with both parents ensures proper supervision and monitoring of children. Parental monitoring has been identified as a protective factor against early sexual debut [43]. Children with Internet access had higher odds of initiation of early sex. This finding does not support previous studies in Ghana, which found Internet access as a protective factor against early sex debut [23]. However, the Internet has been identified as a significant source of sexually explicit materials [1, 51, 52], and there is an association between exposure to sexually explicit materials and early sexual debut [31, 33].

This study found that early sexual debut increases the risk of unwanted pregnancies and their associated consequences, such as termination of education and induced abortion. Also, early sexual debut adversely impacts pregnant children’s wellbeing, makes children disrespectful, and increases their risk of multiple lifetime sexual partners and its associated risks, such as STIs. These findings support previous studies that found that early sexual initiation has detrimental effects on children, including making them disrespectful, leading to multiple lifetime sexual partners, exposing them to STIs, unexpected pregnancies, risky abortions, and interfering with their academic progress [53–55].

### Strengths and limitations of the study

There are some limitations associated with this study. The study employed a cross-sectional design, which limits its ability to establish causality. Furthermore, the data collected from the respondents was based on self-reporting, which introduces the potential for social desirability and recall bias to influence their responses. However, notable strengths of this study include its large sample size and its being nationally representative.

## Conclusion

The prevalence of early sexual debut among children aged 8 to 17 was a little more than one in ten (13.2%), and it is more predominant among female children than male children. Factors causing early sexual debut include trying to engage in sex after watching pornography, self-desire to have sex, being influenced by alcohol consumption, transactional sex for financial or academic assistance, and marital status.

On the one hand, female children, children with tertiary, SHS/Voc./Tech./Com., and JHS education, children who belonged to other religion category and those who are Moslems, children who lived with the father alone, mother alone and non-parents, and children who had access to the Internet had higher odds of initiating sex early. On the other hand, children aged 8 to 10 and 11 to 13, unmarried children, and children who resided in the Northern and Middle ecological zones had lower odds of initiating sex early.

Furthermore, early sexual debut increases children’s risk of unwanted pregnancy, which leads to the termination of children’s education or induced abortion. Also, early sexual debut had adverse impacts on the wellbeing of pregnant children and increased children’s risk of multiple lifetime sexual partners. The consequences of early sexual debut among children calls for policymakers to design appropriate interventions, considering the socio-demographic characteristics of children, to curb its occurrence in Ghana.

## Data Availability

The raw data for the findings of this study are freely available from the corresponding author upon request.

## Abbreviations

AOR: Adjusted Odds Ratio
C.I.: Confidence Interval
FGDs: Focus Group Discussions
JHS: Junior High School
KII: Key Informant Interviews
MoGCSP: Ministry of Gender, Children and Social Protection
RC: Reference Category
SHS: Senior High School
